# Identification of a novel immunological epitope on Hexon of fowl adenovirus serotype 4

**DOI:** 10.1186/s13568-021-01309-2

**Published:** 2021-11-20

**Authors:** Jingyi Liu, Nan Mei, Yaling Wang, Xinjin Shi, Hongjun Chen

**Affiliations:** Shanghai Veterinary Research Institute, Chinese Academy of Agricultural Sciences, 200241 Shanghai, China

**Keywords:** Fowl adenovirus, Hexon protein, Monoclonal antibody, Linear epitope

## Abstract

Fowl adenovirus serotype 4 (FAdV-4), the causative agent of hepatitis-hydropericardium syndrome (HHS), distributed widely in the poultry farms in China. Hexon is one of the major capsid proteins associated with viral species or serotypes. However, the epitopes of Hexon protein remain largely unknown. In this study, a monoclonal antibody (mAb) specific to Hexon protein of FAdV-4, designated as 3G8, was generated. Subsequently, the linear peptide recognized by 3G8 was mapped and identified as ^213^AYGAYVK^219^ using a series of overlapping peptides generated from Hexon protein. Amino acid sequence analysis revealed that the epitope recognized by 3G8 was highly conserved across all the FAdVs. The epitope was immunogenic and could be recognized by FAdV-4 positive chicken serum samples. These findings will enrich our knowledge regarding the epitope on Hexon and provide valuable information for further characterization of the antigenicity of Hexon protein.

## Introduction

Fowl Adenoviruses (FAdVs), belonging to genus *Aviadenovirus*, is a common avian disease distributed worldwide. FAdVs are currently classified into five species (A-E) with 12 serotypes (FAdV-1 to 8a, and -8b to 11) (Meulemans et al. 2001; Niczyporuk 2016). FAdVs infect mainly broilers aged 3-5 weeks and can be transmitted by both fecal-oral route and embryonated eggs (Gunes et al. 2012; Pan et al. 2017). FAdVs can cause hepatitis-hydropericardium syndrome (HHS), inclusion body hepatitis (IBH), respiratory tract disease, and gizzard erosion in chickens (Gjevre et al. 2013; McFerran and Smyth 2000; Niu et al. 2018).

FAdV-4 infection was first reported in the chicken farms in Pakistan in 1987 (Anjum et al. 1989) and soon spread to some regions of Asia and south America (Choi et al. 2012; Dahiya et al. 2002; Toro et al. 1999). However, no widespread epidemics occurs until the outbreak of FAdV-4 in the chicken farms in China in 2015 (Niu et al. 2016; Zhang et al. 2016). Many poultry farms were affected and suffered from great economic losses due to the robust transmission and high pathogenicity of FAdV-4. In addition to FAdV-4, identification of other serotypes of FAdVs such as FAdV-1, FAdV-8a, FAdV-8b, FAdV-10 has been reported in China (Chen et al. 2019, 2020; Cui et al. 2020; Huang et al. 2019; Lv et al. 2021; Zhang et al. 2019). A recent study investigated the specific FAdV in the poultry farms in central China during 2015-2018. The predominant serotype was found to be FAdV-4 which was in 48 isolates, whereas FAdV-10 was found in 24 isolates (Cui et al. 2020). Another epidemiological study investigated 96 poultry farms distributed in 15 provinces in China during 2015–2018. The infection of FAdV-4 was found to be 79.4 % (123/155), while infection of FAdV-8a and 8b is 13.5% (21/155) and 3.9% (6/155), respectively (Chen et al. 2019).

Fowl adenoviruses (FAdVs) are non-enveloped double-stranded DNA viruses. The genome size of FAdV-4 is approximately 45 kb. It encodes three major structural proteins, Hexon, penton base and fiber proteins, which constitute the viral capsid and determine the size of virus particles (Anjum et al. 1989; Kurachi et al. 2007). Antibodies against Hexon protein were produced to distinguish different serotypes of FAdVs (McFerran and Adair 1977) and it has been successfully used in identifying some of the 12 serotypes of FAdVs (Ganesh et al. 2001). Although Hexon is the major determinant of viral serotypes, there has been only one study on the Hexon epitopes in which ^128^PLAPKESMFN^137^ for all species FAdVs and two FAdV-C-specific epitopes ^174^KISGVFPNPNQG^185^ and ^270^DYDDYNIGTT^279^ were identified (Pan et al. 2018). The epitope on Hexon protein remains largely unknown. In this study, a novel monoclonal antibody (mAb), designated as 3G8, against Hexon protein of FAdV-4 was generated and used to screen for epitope. The identified novel linear epitope was immunogenic and was conserved across all the FAdVs. These findings provide valuable information for further characterization of the antigenicity of Hexon protein.

## Materials and methods

### Cells, viruses and serum samples

The chicken liver Hepatocellular carcinoma cell line (LMH) was purchased from the American Type Culture Collection (ATCC) and cultured in DMEM/F12 (Gibco, NY, USA) supplemented with 10% fetal bovine serum (FBS) (Gibco). The murine myeloma SP 2/0 cell line (ATCC, CRL-1581) was cultured in RPMI-1640 medium (Gibco), supplemented with 10% FBS, 100 U/ml penicillin, 100 µg/ml streptomycin and 100 µg/ml fungizone, at 37 °C in a humidified atmosphere of 5% CO_2_.

FAdV-4 strain JS7 was isolated from chickens in poultry farm in Jiangsu province, China. The full-length of strain JS7 was sequenced and accession number was KY436519 in GenBank (Wang et al. 2016). FAdV-1 (ATCC^®^VR-432™), FAdV-10 (ATCC^®^VR-834™), were purchased from ATCC and maintained in Prof. Ye Jianqiang’s lab (Yangzhou University, China).

Chicken sera against FAdV-4 were collected every week from the 14-day-old SPF chickens immunized with inactivated FAdV-4 strain JS7 for a total of 7 weeks. Twenty-two serum samples were collected from chicken infected with FAdV-4. Ten healthy sera samples were collected as negative control.

### Cloning and protein expression

A fragment from 1 to 879 bp of *hexon* gene was amplified from FAdV-4 strain JS7 with a primer of *hexon-1 F* and *hexon-879R* (Table 1) by Polymerase chain reaction (PCR). The obtained PCR fragment was subcloned into pET30a (+) and pcDNA3 with restriction sites of *Bam*HI/*Hin*dIII, respectively. The constructed plasmids, pET30a (+)-hexon879 and pcDNA3-hexon879, were further confirmed by Sanger sequencing. To obtain Hexon peptide, pET30a (+)-hexon879 was transformed into *E. coli* BL21 (DE3) strain and protein expression was induced by 1 mM isopropyl β-D-1-thiogalactopyranoside (IPTG) overnight at 37 °C. The peptide was purified by Ni-NTA resin (Merck Millipore, Temecula, CA) and quantified by a BCA protein assay kit (Thermo Fisher Scientific Inc., Hudson, NH). The purified peptide was stored at − 80 °C until use.

**Table 1:** Primers for full-length and truncated fragments of *hexon*

Primers	Sequence of primers (5′–3′)	Restriction sites
*hexon-1 F*	CATGGATCCATGGCGGCCCTCACGC	*Bam*HI
*hexon-601 F*	CATGGATCCATGGTGCTCGGTCGCTTC	*Bam*HI
*hexon-879R*	CATAAGCTTTTAGAAGTTATCCCTGAACCCGAT	*Hin*dIII
*hexon-798R*	CATAAGCTTTTACGGCACGACTATGGTATCTGG	*Hin*dIII
*hexon-750R*	CATAAGCTTTTAGACGGCCACCGCTCCC	*Hin*dIII
*hexon-657R*	CATAAGCTTTTACTTGACGTAGGCACCGTAAGCG	*Hin*dIII
*hexon-636R*	CATAAGCTTTTAGTAATTGTACTGAGACTTGGC	*Hin*dIII
*hexon-615R*	CATAAGCTTTTAGAAGCGACCGAGCACG	*Hin*dIII
*hexon-600R*	CATAAGCTTTTAGCCGGTGTTGGCGTTTTG	*Hin*dIII
*hexon-582R*	CATAAGCTTTTATACCCGTCGCAGAGGATT	*Hin*dIII
*hexon-570R*	CATAAGCTTTTAAGGATTTCTTCCGGGTCC	*Hin*dIII
*hexon-540R*	CATAAGCTTTTAGGGGAAGACGCCGGAAATC	*Hin*dIII
*hexon-459R*	CATAAGCTTTTACTGACCGGAGGCGGACAC	*Hin*dIII
*hexon-405R*	CATAAGCTTTTACATGGACTCCTTGGGAGCCA	*Hin*dIII
*hexon-375R*	CATAAGCTTTTAAGCCGTGCCGCAGTAGG	*Hin*dIII
*hexon-312R*	CATAAGCTTTTAGGTCGACCCCATGTCCAGG	*Hin*dIII

### Generation of mAb

The 6-week-old female BALB/C mice were intraperitoneally immunized with purified Hexon peptide as described previously (Wang et al. 2018). At 3 days post the last immunization, the immunized mice were euthanized for the collection of spleen cells. The collected spleen cells were fused with SP 2/0 myeloma cells as described (Wang et al. 2018). Antibody specific to Hexon protein secreted from the hybridoma cells were screened by IFA. Following three times subclone of the positive hybridoma cells, 1 × 10^5^ cells were used to injected mice for mAb production and collection. All animal experiments were carried out strictly following the guidelines for animal use with approval from Shanghai Laboratory Animal Management Committee and the Animal Care and Use Committee of Shanghai Veterinary Research Institute, Chinese Academy of Agricultural Sciences (permit number: SYXK 2020-0027).

### IFA assay

LMH cells were transfected with the constructed plasmid, pcDNA3-Hexon879, or infected with FAdV-4, FAdV-1 and FAdV-10, respectively. At 70 h post-transfection, the medium was discarded and cells were fixed with 4% paraformaldehyde for 20 min. Following three washes with tris-buffered saline (TBS) containing 0.5% Tween-20 (TBST), the cells were fixed with cold acetone and ethanol (3:2) for 5 min. Then, mAb 3G8 diluted at 1:1000 was added to the cells and incubated for 30 min at room temperature. The cells were washed three times with TBST and incubated with FITC-conjugated goat anti-mouse antibody diluted at 1:600 (Molecule probe) for 30 min at RT. Then, the cells were washed three times with PBS and examined under an Axiophot Photo microscope (Carl Zeiss).

### Mapping of epitope of Hexon recognized by mAb 3G8

To identify the antigenic epitope of Hexon protein recognized by mAb 3G8, a number of truncated Hexon fragments were amplified using primers listed in Table 1. Briefly, N terminal truncated fragments of hexon879 were amplified by PCR with primers of *hexon-1 F* and *hexon-879R* for hexon879 (1–879 bp), and then various reverse primers were designed to amplify the truncated fragments as below: *hexon-798R* for hexon798 (1–798 bp), *hexon-750R* for hexon750 (1–750 bp), *hexon-657R* for hexon657 (1–657 bp), *hexon-636R* for hexon636 (1–636 bp), *hexon-615R* for hexon615 (1–615 bp), *hexon-600R* for hexon600 (1–600 bp), *hexon-582R* for hexon582 (1–582 bp), *hexon-570R* for hexon570 (1–570 bp), *hexon-540R* for hexon540 (1–540 bp), *hexon-459R* for hexon459 (1–459 bp), and *Hexon-405R* for hexon408 (1–405 bp), *hexon-375R* for hexon375 (1–375 bp), *hexon-312R* for hexon312 (1–312 bp), hexon601-879 fragment was amplified with *hexon601F* and *hexon-879R*., Amplified fragments were cloned into pET-30a(+), respectively. Following confirmation by Sanger sequencing, the constructed plasmids were transformed into BL21 (DE3) competent cells for protein induction as previously describe.

### Western blot

The expressed truncated Hexon proteins were analyzed by SDS-PAGE and transferred onto nitrocellulose (NC) membrane for Western blot. After blocking with 5% skimmed milk in TBST for 2 h at room temperature, the membrane was incubated with mAb 3G8 diluted at 1:1000 in TBST at 37 °C for 1 h. Following three washes with TBST, the membrane was incubated with HRP-conjugated goat anti-mouse antibody diluted at 1:5000 at 37 °C for 1 h. The result was visualized by Pierce™ Supersignal West Pico Chemiluminescent Substrate (Thermo) using chemiluminescence image analysis system (Tanon 5200).

### Sequence analysis of the epitope

The amino acid sequence of FAdV-4 strain JS7 containing the identified epitope was compared with those in different serotypes of FAdVs. The sequences used for alignment are FAdV-4 strain JS7(AUD07657.1), FAdV-4 strain HLJFAd15 (APA19522.1), FAdV-4 strain ON1 (ADQ39061.1), FAdV-1 strain CELO (AAC54912.1), FAdV-2 strain SR48 (ANJ02381.1), FAdV-3 strain SR49 (ANJ02418.1), FAdV-5 strain 340 (YP_007985654.1), FAdV-6 strain CR119 (ANJ02455.1), FAdV-7 strain YR36 (ANJ02492.1), FAdV-8a strain TR59 (ANJ02529.1), FAdV-8b strain 764 (ANJ02566.1), FAdV-9 strain A-2 A (NP_050287.1), FAdV-10 strain C-2B (ALE15153.1), and FAdV-11 strain 380 (ANJ02603.1). The sequence alignment was performed by ClustalW Multiple Alignment.

### Reactivity of the synthetic peptide with chicken serum samples

The 96-well plate was coated with 1 µg of synthesized epitope peptide in carbonate-bicarbonate buffer (pH 9.6) per well at 4 ºC overnight. The next day, the plate was washed with PBS containing 0.1% tween 20 (PBST) for three times, followed by blocking the plate with 5 % skimmed milk in PBST at 37 ºC for 1 h. The plate was washed with PBST three times and incubated with chicken serum samples (diluted at 1:100) individually at 37 ºC for 1 h. The plate was washed with PBST three times and incubated with HRP-conjugated goat anti-mouse secondary antibody (diluted at 1:5000) at 37 ºC for 1 h. Then, the plate was washed with PBST for three times and 100 µL 3,3′,5,5′-tetramethyl benzidine dihydrochloride (TMB) was added to each well. The reaction was stopped 10 min later by adding 50 µL of 2 M sulfuric acid (H_2_SO_4_) per well. The absorbance was determined using a microplate reader (BioTek, USA) at 450 nm. The cutoff value was computed as 2.1 times of the mean absorbance values of the chicken negative sera.

## Results

### Generation of a novel mAb against Hexon protein

**Fig. 1:**
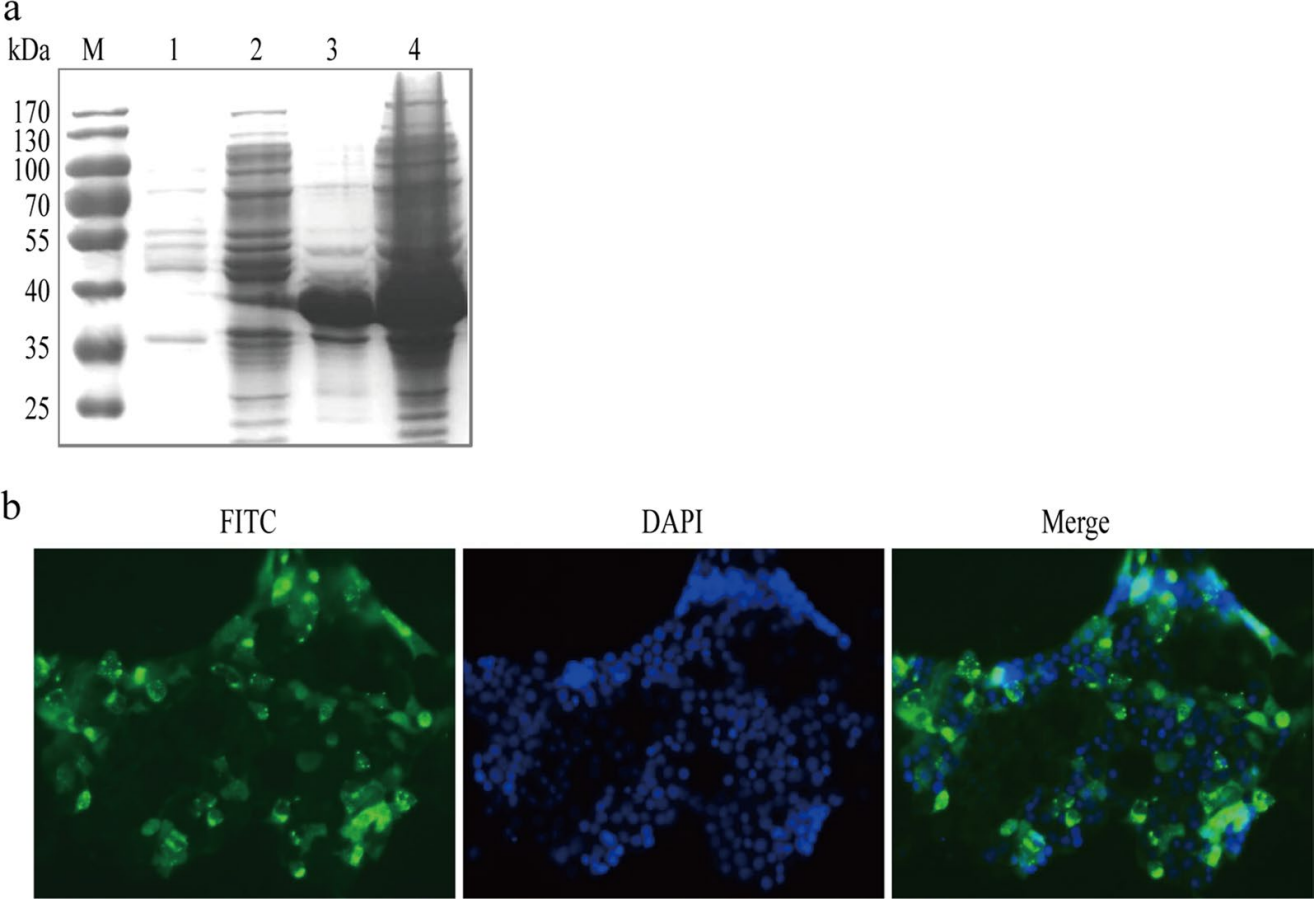
Characterization of the generated mAb 3G8. **a** The N-terminus of Hexon peptide (1-293aa) of FAdV-4 was expressed and analyzed by SDS-PAGE. 1, empty vector; 2, non-induced BL21 (DE3) transformed with pET30a (+)-hexon879; 3, purified Hexon protein; 4, induced BL21 (DE3) transformed with pET30a (+)-hexon879. **b** The mAb 3G8 reacted with the Hexon protein expressed in cell by IFA. pcDNA3-hexon879 plasmid was transfected to LMH cells. At 70 h post-transfection, the cells were fixed and incubated with mAb 3G8 for 30 min. Following three washes with PBS, the cells was incubated with FITC-conjugated anti-mouse antibody and examined under an Axiophot Photo microscope

The Hexon peptide was successfully expressed, purified and analyzed by SDS-PAGE (Fig. 1a). The purified Hexon protein was used to immune Balb/c mice for antibody production. A hybridoma cell line secreting mAb, designated as 3G8, was generated. IFA analysis showed that the LMH cells transfected with the constructed plasmid, pcDNA3-Hexon879, could be recognized by the generated mAb (Fig. 1b).

### Identification of antigenic epitope in Hexon recognized by mAb 3G8

**Fig. 2:**
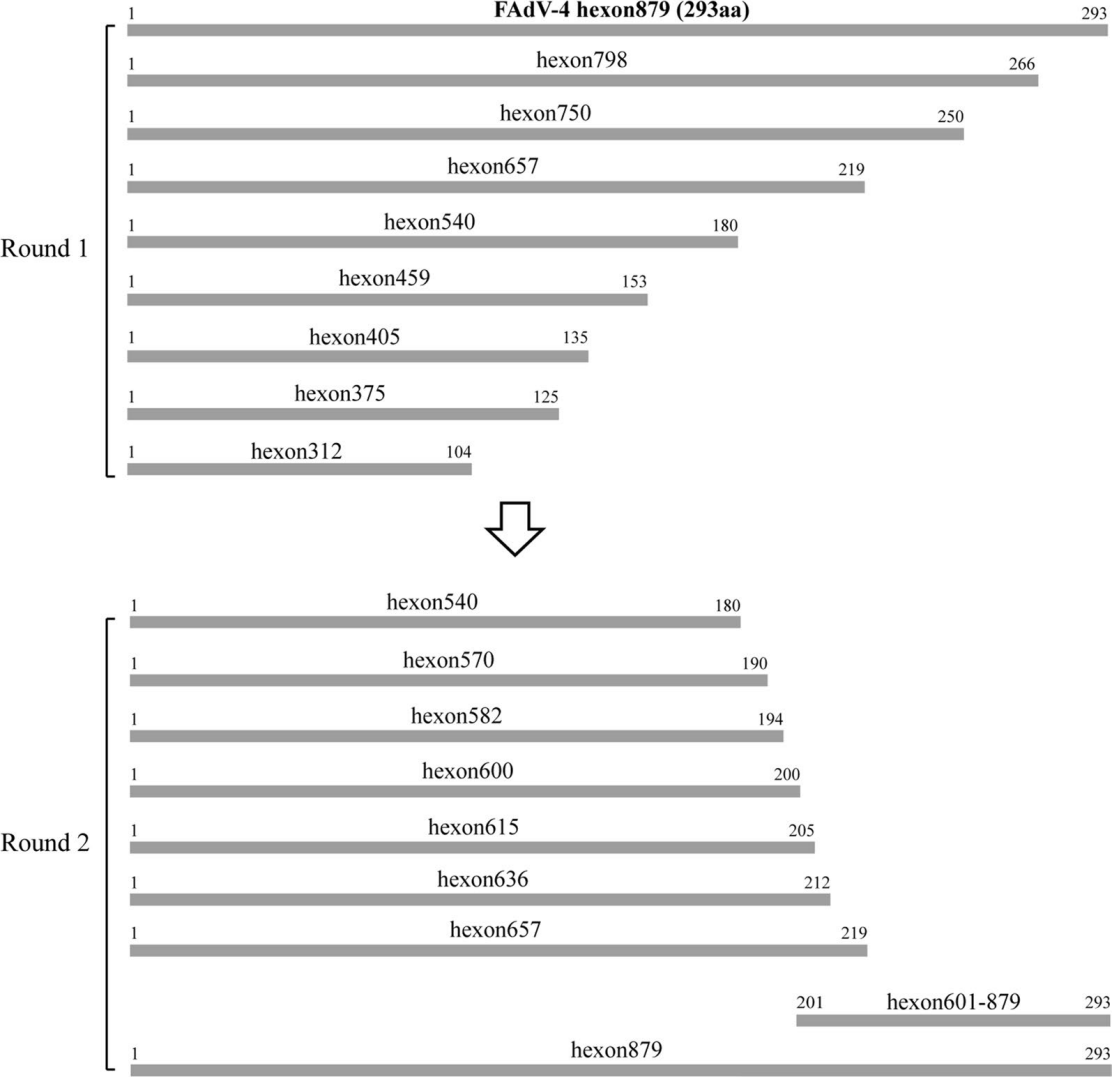
Schematic representation of FAdV-4 Hexon fragments used for epitope mapping. Two rounds of truncated Hexon proteins were conducted to investigate the epitope of the generated mAb

**Fig. 3:**
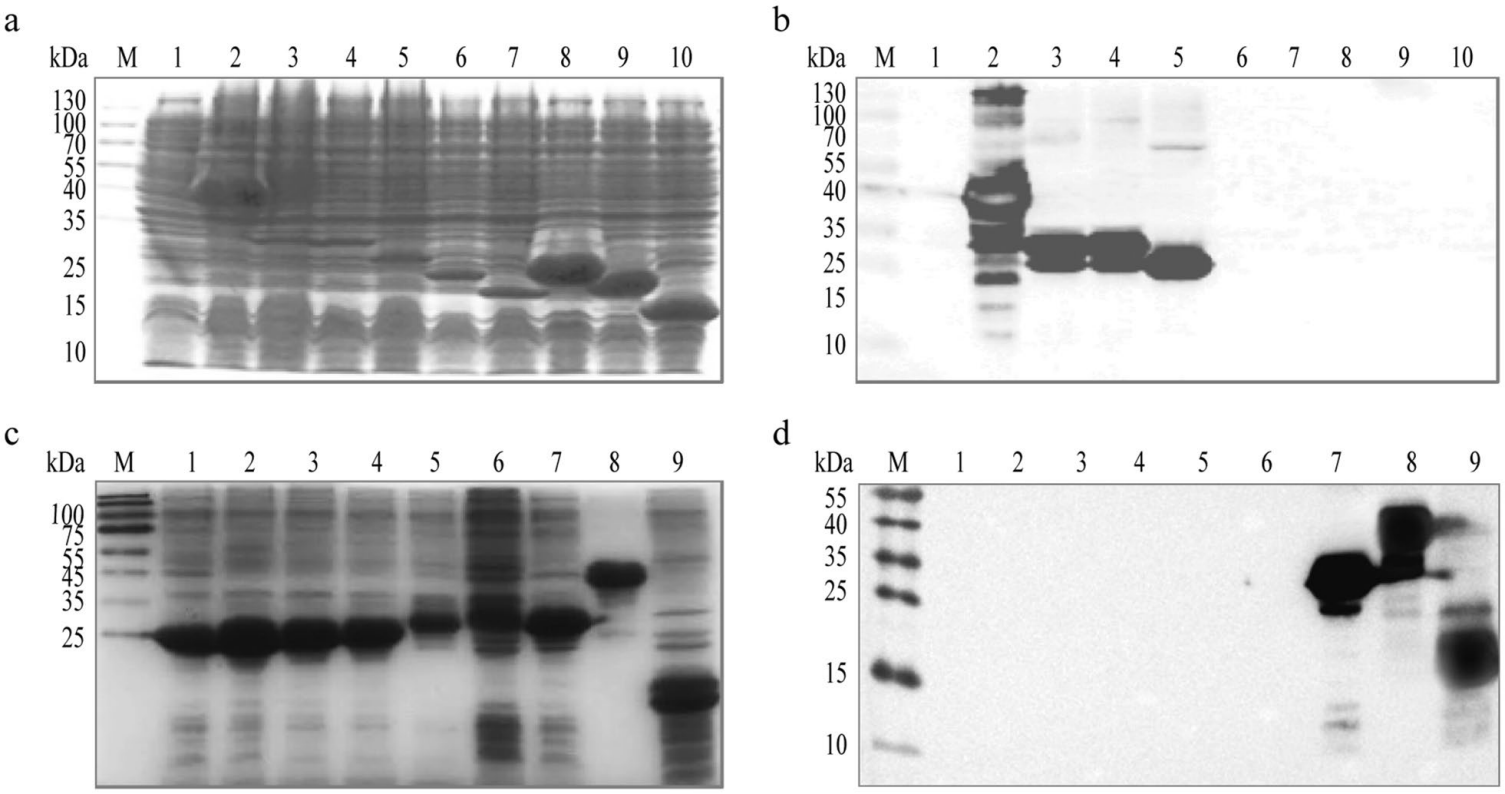
Identification of antigenic epitope in Hexon of FAdV4 recognized by mAb 3G8. SDS-PAGE (**a**) and WB (**b**) analysis of the expression of truncated Hexon proteins. A series of *hexon* fragments were cloned into pET-30a (+) and expressed in *E. coli*. Antigenic epitope of mAb 3G8 was located between hexon657 and hexon540. 1, empty vector;2, hexon879; 3, hexon798;4, hexon750; 5, hexon657; 6, hexon540; 7, hexon375; lane 8, hexon459; lane 9, hexon405; 10, hexon312. SDS-PAGE (**c**) and WB (**d**) analysis of the expression of truncated Hexon proteins. A series of *hexon* fragments were cloned into pET-30a (+) and expressed in *E. coli*. An epitope of “AYGAYVK” located between hexon636 and hexon657 was characterized as the antigenic epitope recognized by mAb 3G8. 1, hexon540; 2, hexon570; 3, hexon582; 4, hexon600; 5, hexon615; 6, hexon636; 7, hexon657; 8, hexon879; 9, hexon601-879.

To determine the antigenic peptide of Hexon recognized by mAb 3G8, two rounds of overlapping peptides were designed (Fig. 2) and expressed in BL21 (DE3) (Fig. 3a, c). For the first round, the mAb 3G8 could robustly react with hexon879, hexon798, hexon750, and hexon657, but not with hexon540, indicating the linear epitope recognized by mAb 3G8 is located between 541 nt and 657 nt (Fig. 3b). To further identify the exact linear epitope, the region from 541 nt to 879 nt was designed to generate 6 more truncated Hexon proteins, designated as hexon636, hexon615, hexon600, hexon582, hexon570, and hexon601-879. Western blot analysis showed that mAb 3G8 could react with hexon657, hexon601-879, but not with hexon636, suggesting that the linear epitope recognized by mAb 3G8 is located between 637 nt and 657 nt (Fig. 3d). Sequence analysis revealed that the amino acid of the identified linear epitope is ^213^AYGAYVK^219^.

### Amino acid alignment of the identified epitope among FAdVs

**Fig. 4:**
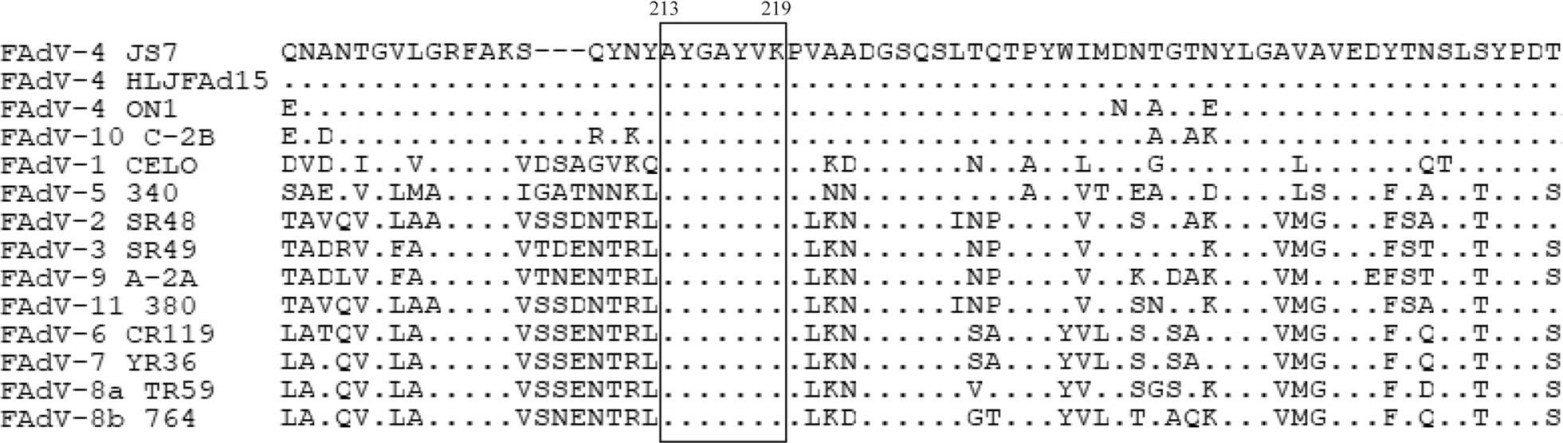
Amino acid alignment of the epitope in Hexon of FAdVs. The amino acid sequence of FAdV-4 JS7 containing the identified epitope was compared with those in 12 serotypes of FAdVs. The sequences used here are FAdV-4 strain JS7 (AUD07657.1), FAdV-4 strain HLJFAd15 (APA19522.1), FAdV-4 strain ON1 (ADQ39061.1), FAdV-1 strain CELO (AAC54912.1), FAdV-2 strain SR48 (ANJ02381.1), FAdV-3 strain SR49 (ANJ02418.1), FAdV-5 strain 340 (YP_007985654.1), FAdV-6 strain CR119 (ANJ02455.1), FAdV-7 strain YR36 (ANJ02492.1), FAdV-8a strain TR59 (ANJ02529.1), FAdV-8b strain 764 (ANJ02566.1), FAdV-9 strain A-2 A (NP_050287.1), FAdV-10 strain C-2B (ALE15153.1), and FAdV-11 strain 380 (ANJ02603.1). The identified epitope was shown in box.

Amino acid alignment was performed to evaluate the conservation of the identified epitope among different strains and serotypes of FAdVs (Fig. 4). The epitope (^213^AYGAYVK^219^) was highly conserved across all the FAdVs.

### Epitope recognized by mAb 3G8 is conserved across FAdVs

**Fig. 5:**
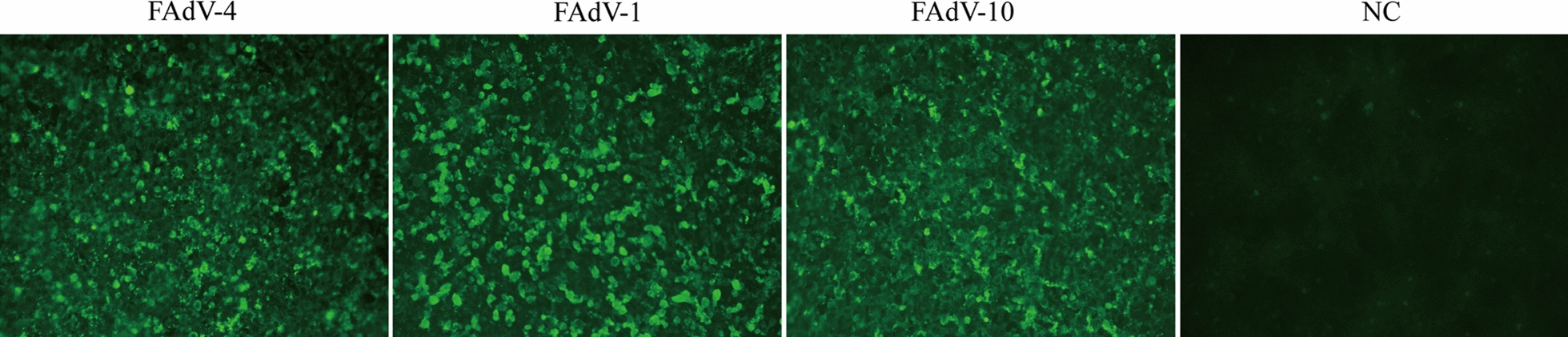
Reactivity of the mAbs 3G8 with different serotypes of FAdVs by IFA. LMH cells were infected with FAdV-4, FAdV-1 and FAdV-10, respectively. At 70 h post-infection, the cells were incubated with mAb 3G8 and detected by IFA, respectively. LMH cells treated with PBS was used as negative control. mAb 3G8 was able to recognize all three serotypes of FAdVs

To confirm the amino acid alignment results, we evaluated the reaction of mAb 3G8 with different serotypes of FAdVs. LMH cells were infected with FAdV-1, FAdV-4 and FAdV-10, respectively. At 70 h post-infection, the cells were incubated with mAb 3G8 followed by a secondary antibody. LMH cells treated with PBS was used as control. It was shown that mAb 3G8 can recognize FAdV-1, FAdV-4 and FAdV-10, which corresponded with the amino acid sequence analysis (Fig. 5). The data suggested that the antibody is conserved in these serotypes of FAdVs.

### Reactivity of the synthetic peptide with chicken serum samples

**Fig. 6:**
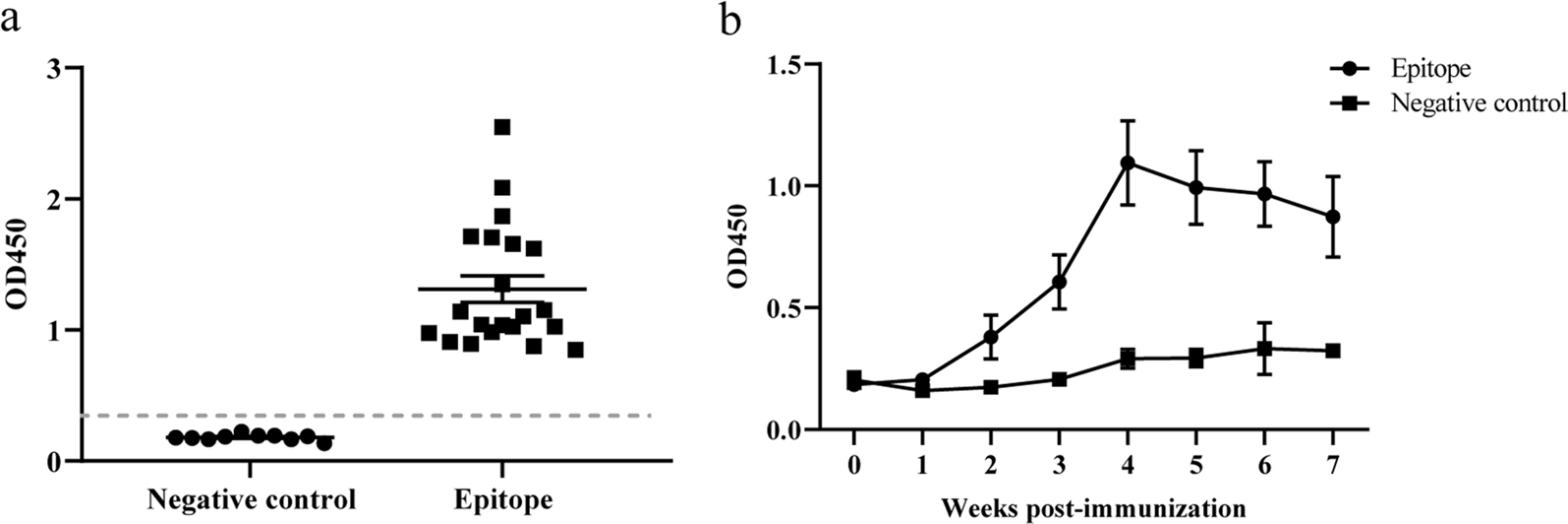
Reactivity of the epitope with chicken serum samples. The immunogenicity of the identified epitope was investigated using serum samples from FAdV-4 infected chickens (**a**) and inactivated FAdV-4 immunized chickens (**b**). The cut-off was set as 2.1 times the mean OD_450_ value of the ten healthy chicken serum samples

Twenty-two positive sera samples collected from FAdV-4 strain JS7 infected chickens and ten negative sera samples were used to assess whether the epitope is immunogenic. As the mean of the OD_450_ value of the negative samples was 0.1804, the cut-off was defined as 2.1 times the mean OD_450_ value of the negative samples. Therefore, the OD_450_ for the cut-off of the ELISA assay was determined as 0.3788. The result showed that all twenty-two positive serum samples were able to recognize the peptide (Fig. 6a). Moreover, the serum sample of chicken at 2 weeks post-immunization was able to recognize the epitope (Fig. 6b). These results indicated that the identified epitope was associated with chicken immune response during FAdVs infection.

## Discussion

HHS, caused by FAdV-4, is a severe avian disease distributed widely and has leaded to significant economic losses to the poultry industry in China. Hexon is the major capsid protein of fowl adenoviruses and contains virus-neutralizing activity and serotype specificity (Matsushima et al. 2011; Roberts et al. 2006). Hexon has been widely used as target to study the molecular epidemiology due to its antigenic determinants. Identification of Hexon epitope plays an important role in understanding its antigenic characteristics. However, little is known about the epitope on the Hexon protein of FAdVs. To better understand the antigenicity of Hexon protein, N terminus (N293aa) of Hexon protein of FAdV-4 strain JS7 was expressed, purified in this study. A novel mAb was generated and the epitope on Hexon recognized by the mAb was further analyzed.

Although Hexon is the major capsid protein that determines viral serotypes, very few epitopes on Hexon protein of FAdVs were reported. In a previous study, three B cell epitopes, ^128^PLAPKESMFN^137^, ^174^KISGVFPNPNQG^185^ and ^270^DYDDYNIGTT^279^ on Hexon of FAdV-4 strain HLJFAd15 were identified using two mAbs generated by the L1-Hexon protein of FAdV-4 strain HLJFAd15 (Pan et al. 2018). All the three epitopes were highly conserved in FAdV-C species, including FAdV-4 strain ON1, FAdV-4 strain JS7, and FAdV-10 strain C-2B. ^128^PLAPKESMFN^137^ was relatively conserved across all the FAdVs species with three mutations appeared in the other four species, while ^174^KISGVFPNPNQG^185^ and ^270^DYDDYNIGTT^279^ were poorly conserved between FAdV-C and the other four species. Here, based on the generated mAb 3G8, a novel antigenic epitope AYGAYVK located between 213 aa to 219 aa on Hexon was identified. Amino acid alignment showed that the identified epitope was highly conserved across all the species of FAdVs. IFA analysis of the activity of mAb 3G8 against different serotypes of FAdVs has confirmed that the identified epitope is conserved across FAdV-1, FAdV-4 and FAdV-10. To further investigate whether the identified epitope is immunogenic, the reactivity of the synthesized epitope with chicken serum samples was investigated. The result demonstrated that FAdV-4 positive chicken serum was able to recognize the identified epitope.

In summary, a novel mAb against Hexon of FAdV-4 was generated and applied to identify its antigenic epitope. The domain of ^213^AYGAYVK^219^ was identified as a new immunological epitope and was found to be highly conserved across all the serotypes of FAdVs. The identified epitope could be recognized by FAdV-4 positive chicken serum samples. These findings will enrich our knowledge regarding the immunological epitope of Hexon and provide valuable information for further characterization of the antigenicity of Hexon protein.

## Data Availability

The datasets used and/or analyzed during this study are available from the corresponding author on reasonable request.
